# Successful (?) Removal of Spleen

**Published:** 1874-01

**Authors:** 


SUCCESSFUL (?) REMOVAL OF THE SPLEEN.—In the *Raccoglitore Medico*, Dr. Sonsino gives an account of a case in which, on June 20th, Dr. Attillio Urbinato, of Cesino, removed a hypertrophied and mobile spleen. The incision was made in the middle line, and prolonged above the umbilicus, being at least seven inches in length. The operation was performed without much difficulty. After tying three small cutaneous arteries, opening the peritoneum, and drawing aside some loops of intestine, the spleen was seen, free from all abnormal adhesions, and of enormous size. At the inferior part was seen the gastro-splenic epiploon, which was adherent; and the vessels here were extremely dilated. At the upper part was seen the lower portion of the pancreas. The epiploon was detached, and the vessels tied. The ligatures, seven in number, were left inside without further precaution. The few adhesions of the pancreas were overcome without difficulty, simply by means of the finger. The largest vessels, and the connective tissue which surrounded them, were secured by a metallic loop and hempen ligature. The "toilette" of the abdominal cavity was made with great care. The patient lost but little blood. The ligatures of the vessels tied were passed out between the sutures, of which there were five deep and five superficial. The spleen weighed two and a half pounds. The operation lasted an hour; the patient bore the chloroform well, and subsequently appeared to be progressing favorably, but died of peritonitis three days after the operation.—*British Medical Journal.*

